# Use of surgical task shifting to scale up essential surgical services: a feasibility analysis at facility level in Uganda

**DOI:** 10.1186/1472-6963-13-292

**Published:** 2013-08-01

**Authors:** Moses Galukande, Sam Kaggwa, Patrick Sekimpi, Othman Kakaire, Achilles Katamba, Ian Munabi, Francis Mwesigye Runumi, Ed Mills, Amy Hagopian, Geoffrey Blair, Scott Barnhart, Sam Luboga

**Affiliations:** 1 Department of Surgery, College of Health Sciences, Makerere University, P.O. Box 7072, Mulago Hill Road, Kampala, Uganda; 2 Department of Orthopaedics, College of Health Sciences, Makerere University, Kampala, Uganda; 3 Department of Obstetrics & Gynaecology, College of Health Sciences, Makerere University, Kampala, Uganda; 4 Clinical Epidemiology Unit, College of Health Sciences, Makerere University, Kampala, Uganda; 5 Department of Anatomy, College of Health Sciences, Makerere University, Kampala, Uganda; 6 Ministry of Health, Kampala, Uganda; 7 University of Washington, Seattle, USA; 8 University of British Columbia, Vancouver, Canada; 9 University of Ottawa, Ottawa, Canada

**Keywords:** Surgical, Task shifting, Uganda, Human Resource for Health crisis

## Abstract

**Background:**

The shortage and mal-distribution of surgical specialists in sub-Saharan African countries is born out of shortage of individuals choosing a surgical career, limited training capacity, inadequate remuneration, and reluctance on the part of professionals to work in rural and remote areas, among other reasons. This study set out to assess the views of clinicians and managers on the use of task shifting as an effective way of alleviating shortages of skilled personnel at a facility level.

**Methods:**

37 in-depth interviews with key informants and 24 focus group discussions were held to collect qualitative data, with a total of 80 healthcare managers and frontline health workers at 24 sites in 15 districts. Quantitative and descriptive facility data were also collected, including operating room log sheets to identify the most commonly conducted operations.

**Results:**

Most health facility managers and health workers supported surgical task shifting and some health workers practiced it. The practice is primarily driven by a shortage of human resources for health. Personnel expressed reluctance to engage in surgical task shifting in the absence of a regulatory mechanism or guiding policy. Those in favor of surgical task shifting regarded it as a potential solution to the lack of skilled personnel. Those who opposed it saw it as an approach that could reduce the quality of care and weaken the health system in the long term by opening it to unregulated practice and abuse of privilege. There were enough patient numbers and basic infrastructure to support training across all facilities for surgical task shifting.

**Conclusion:**

Whereas surgical task shifting was viewed as a short-term measure alongside efforts to train and retain adequate numbers of surgical specialists, efforts to upscale its use were widely encouraged.

## Background

The poor availability of surgical services in developing countries is a long neglected problem that has recently gained attention [1,2]. Violence, injury, and obstetric emergencies are among leading causes of mortality and morbidity that can be mitigated through surgical intervention [3]. Surgical interventions are often viewed as expensive and complex, but many common problems amenable to surgery in resource-limited settings are cost-effective and do not require specialized staff and equipment [4].

One of the main barriers to surgical care--defined as the safe provision of preoperative, operative, and post-operative surgical and anesthesia services--in resource-limited settings is the shortage of trained health workers. Africa accounts for 24% of the global disease burden but enjoys only 3% of the global health workforce [5]. In Uganda there are only approximately 100 general surgical specialists for nearly 33 million people [6]. Meanwhile reports of surgical output [3,6] i.e. ratios of operations/population, are exceedingly low in poor countries. Shortages of surgical human resources are partially responsible for this low output.

Surgery is considered a highly specialized field that requires long years of training: in the US, surgeons complete a five-year surgical residency before operating independently. Indications for surgery are not always straightforward, patient management decisions can be complex, and learning the technical skills required to perform major surgery requires committed trainers. All this leads to a concern that such complex skills and knowledge cannot be adequately transferred to less-than-fully-trained surgeons in a shortened training course(s).

Non-specialist physicians in general hospitals and the highest level health clinics (HC-IVs) are presumed to be adequately trained and available to handle emergency and essential surgical care, yet there is a limited scope of life saving surgery conducted at these units. The most commonly performed maternal procedures at district hospitals are cesarean sections and uterine evacuations for obstetrics, while the most common general surgical procedures) are hernia repairs and wound care for trauma (which may include manipulation of fractures [5]. With the advent of mass safe male circumcision for partial HIV prevention, circumcision also will be a commonly-performed surgical procedure [7,8]. These can be safely managed by non-surgeons. Highly complicated procedures that require the expertise of fully trained surgical specialists can then be referred to tertiary Hospitals. Task shifting holds a great promise to contribute to alleviation of Human Resource for Health crises [9,10].

The purpose of this study was to collect the views of health facility managers and clinicians on surgical task shifting. This paper focuses on the qualitative assessment of facility staff views of feasibility, practicality and advisability of such task-shifting. We conducted this assessment as a prelude to designing an intervention to build surgical skills for non surgical physicians (NSPs), under a task-shifting grant from the government of Canada.

## Methods

Our mixed-method study was conducted in 24 purposively and randomly selected facilities; eighteen general hospitals and six health centre IVs from a list of more than 120 Ugandan hospital and health centers made available from the Ministry of Health. The qualitative methods included one focus group discussion in each of the 24 facilities, and 37 key informant interviews (KIIs), one or two in each facility. Interviews were conducted with health workers and heads of units at these facilities and heads of units. We used a standard set of focus group and interview questions.

The purpose of the focus group discussions and interviews was to obtain health workers’ understanding, experiences, and views of surgical task shifting. A multidisciplinary team, comprising a social scientist and one or two surgeons met with a mixed-cadre group of 6–8 health workers from the units studied. A pre-prepared interview guide was followed along particular themes including: understanding, practice, acceptability and perceived barriers to surgical task shifting. The proceedings were captured using a voice recorder, this was backed up by taking notes.

Respondents were purposively selected to include those involved in the provision of surgical care services in the facilities visited. However, other cadre levels were represented in focus group discussions and key informant interviews.

We asked for the definition of task-shifting, how it manifested, rationale for its practice, the acceptability, the procedures involved, when it is appropriate, the perceived barriers to task shifting it, for how long it should go on, its effect on patient outcomes and any recommendations.

All respondents were informed of the purpose, risks, and benefits of participation in the discussions, and written informed consent was obtained from all. Discussions were audio-taped and transcribed verbatim, by the original interviewer.

The discussions were conducted in English at quiet locations on the premises of the health units, and lasted 60 to 90 minutes. IRB approval from Makerere University was obtained before this study was started.

Data on the surgical burden of disease, procedures performed and resources available for surgical procedures were collected from the selected facilities using a Standard (pre tested) questionnaire.

In additional to the FGDs and KIIs, we also reviewed the theater operation logs data from 12 hospitals and 2 health centers IVs over a 3 month period November 2009 through January 2010 to examine surgical volumes. We limited this review to half of the facilities originally surveyed due to logistical constraints.

### Data management and analysis

Voice records were transcribed by the original interviewer and harmonized with the notes originally written during the discussions. The transcripts were read in their entirety, and themes were developed through an interactive, collaborative process with the study team, including all of the original interviewers. Matrices were created to track themes across participating health care facilities. The findings under each theme were attached to illustrative quotations and phrases from individual participant’s responses, to illustrate and support the themes. During the analysis, we used numbers to track how many of the 24 focus groups touched on an identified theme.

### Data from theatre records

All surgical theatre logs were photocopied to allow collection of demographic and pertinent care variables, including: diagnosis, procedures performed and category of procedure.

## Results

We conducted 24 focus groups and 37 key informant interviews at 24 facilities. These included 18 hospitals as shown in Table 1 (Gombe, Nkozi, Mityana, Mubende, Kalisizo, Kitovu, Kisubi, Bugiri, Rakai, Iganga, Entebbe, Kayunga, Nyenga, Kawolo, Lyantonde, Villa Maria, Kiboga, and Nakaseke) and 6 Health Centre IVs (St. Stephens, Atirir, Serere, Kakira, Kasana and Wakiso).

**Table 1:** Selected Uganda health facilities (hospitals and HC IVs)

	**Hospital**	**Type**	**Bed capacity**	**District Population/Catchment area**	**Distance from Kampala (Km)**	**Health workers numbers**			
						**Dr**	**CO**	**Nur**	**MW**
1	Bugiri	PH	100	24,800	166	5	-	34	12
2	Gombe	PH	100	100,000	68	5	4	23	13
3	Iganga	PH	120	51,800	205	9	-	61	22
4	Kakira	NGH	100	49,000	100	2	27	2	2
5	Kasana - Luweero	HC IV	150	100,000	86	3	-	9	12
6	Kawolo	PH	110	35,500	45	3	-	-	-
7	Kayunga	PH	150	23,100	74	2	7	25	18
8	Kiboga	PH	120	16,600	132	4	18	50	15
9	Kisubi	NGH	100	50,000	35	4	4	38	16
10	Kalisizo	PH	200	32,700	150	4	8	-	-
11	Mityana	PH	120	266,100	77	4	-	-	-
12	Mubende	PH	100	436,500	170	2	-	-	-
13	Rakai	PH	100	466,300	174	4	7	7	14
14	Nakaseke	GH	120	100,000	65	5	-	-	-
15	Nkozi	NGH	100	100,000	85	2	5	10	7
16	Nyenga	NGH	100	100,000	59	3	5	31	7
17	Villa Maria	NGH	126	500,000	318	6	4	10	-
18	Kitovu	NGH	220	228,200	140	6	5	40	14
19	Wakiso	HC IV	30	50,000	40	1	1	-	-
20	Kabula Lyantonde	HCIV	30	25,000	202	3	5	27	16
21	Kasana luweero	HCIV	30	100,000	86	3	-	9	12
22	St Stephen’s	HCIV	20	100,000	7	1	2	4	4
23	Atitir	HCIV	34	10,300	299	1	2	5	3
24	Serere	HCIV	30	176,500	205	1	2	8	2

*PH* Public Hospital, *HC IV* Health center IV, *GH* General Hospital, *NGH* Non Governmental hospital, *Dr* Doctors, *CO* Clinical Officers, *Nur* Nurses, *MW* Midwives.

- missing data.

Source of data: Uganda task-shifting feasibility study 2012.

The majority of these facilities had a 100–150 bed capacity (Uganda’s Ministry of Health standard for a general hospital capacity is 100 beds). Patient attendance figures indicated an average of 80 outpatient visits per day per site. Other statistics for catchment population and staffing are shown in Table 1.

In Table 2, the scope and the frequencies of the commonly performed procedures for the participating hospitals are shown.

**Table 2:** Scope and frequencies of the surgical procedures commonly performed in selected Uganda health facilities between November 2009 and January 2010

**Procedures**	**Sum (%)**
C- Section	1057 (33)
Hernia repair	683 (22)
Uterine Evacuation	377 (12)
Surgical Toilet & Suture	203 (6.4)
Incision & Drainage	202 (6.4)
Laparotomy (various indications)	194 (6.1)
Circumcision	120 (3.8)
Hysterectomies (STAH/TAH)	71 (2.2)
Urinary Retention relief	54 (1.7)
Superficial skin masses	49 (1.5)
Tubal ligation	38 (1.2)
Hydrocelectomy	33 (1.0)
Closed manipulation of fractures	22 (0.7)
Ano rectal conditions	17 (0.5)
Appendicectomy	11 (0.4)
Cervical Tear repair	11 (0.4)
Sequestrectomy	10 (0.4)
Vaginal Vesico Fistula repairs	7 (0.2)
Breast lump Excision	5 (0.2)
Cervical cancer EUA	3 (0.1)

Source of data: operating room logs of hospitals in the Uganda task-shifting feasibility study 2012.

### Definition of surgical task shifting

On the whole respondents had a good understanding of the concept of surgical task shifting. Most respondents (21/24 focus groups or FGDs) described it as an active or deliberative process of passing on responsibility to a higher to lower cadre person who had not been specifically trained for the task at hand. A few (3/24 FGDs) were not familiar with the term.

Here are some of the definitions stated by the focus group members and key informants:

1) Delegation of work particularly from a person with higher training to one with less training;

2) Performing work one is not trained to do;

3) Performing activities outside one’s mandate;

4) Performing a task on one’s own after recognizing a need; and/or

5) The transfer of tasks among health units i.e. from a health centre to a hospital or from a higher health unit to a lower one.

One respondent, similar to a few others, described surgical task shifting as something almost illicit:

“Doing work which you were not supposed to be doing like performing a Caesarian Section when you are not trained to do it ( for example, a Clinical Officer doing a Caesarian Section)” – FGD, in a Hospital”

Another viewpoint emphasized a delegation of surgical tasks to low-level personnel:

“I think it means shifting a task to someone else when the doctor is not around to perform a surgical procedure”- FGD in a Hospital

Another respondent characterized it as a facility transfer rather than a task transfer:

“It can be defined in two ways, a unit shifting the task to another unit. That is, what work to be done in a particular unit is shifted to a lower unit, for example, from a hospital to a health centre IV”- FGD in a hospital

### Justification for task shifting

Several reasons were given to explain why surgical task shifting took place in the visited health facilities.

Understaffing was the main driver for task shifting, most respondents (17/24 FGDs) said so.

Most respondents (15/24 FGDs) also noted task shifting is done for high patient-load reasons, for example when a higher number of patients need services than the existing number of providers can handle. Some (6/24 FGDs) contended that staff resorted to surgical task shifting when patients refuse to be referred to other hospitals due to failure to afford services at more distant sites the prohibitive costs are involved in accessing services at distant sites are contributed to be transport costs.

Understaffing is the main precipitating factor in all instances, most respondents said.

A number of respondents (10/24 FGDs) said task-shifting should be done “to save lives”, with fewer (8/24 FGDs) mentioning the benefit to health workers of acquiring additional skills or earning extra income, especially in private clinics. Participants in a few groups (5/24) said this approach could reduce patient waiting time, and some (3/24 FGDs) also mentioned the scarcity of specialized surgeons as a major driver.

One participant, similar to many others, said,

“Scarcity of skilled human resource it may not necessarily mean that, it is the lack of numbers but, the professional to perform a particular complex procedure is not around” – FGD in one of the Hospitals

A couple of people (2/24 FGDs) discussed the problems of surgeon absenteeism, while others (in 2/24 FGDs) mentioned the need to reduce preventable referrals, and two (2/24 FGDs) mentioned the shortage of equipment.

“*Long distances hinder accessibility of patients to services, so referrals are not possible, so patients stay here and we work on them” – FGD in a hospital*

### Perceived effects of surgical task shifting

All focus groups included people who were convinced surgical task shifting would decrease mortality because complications could be averted by the timely management of surgical emergencies or the need for urgent intervention.

However, some respondents feared surgical task shifting could increase mortality in instances where incompetent trainees are left unsupervised. Potential pitfalls of surgical task shifting were named; we list them in order of perceived importance:

1. Possible increase in morbidity and mortality

2. Low staff motivation to take on extra load

3. Lack of facilitation, equipment and space

4. Medical- legal responsibility for mishaps

5. Lack of public acceptance for the concept

6. Staff over stepping their boundaries

7. Lack of support supervision

8. Cost of training and support supervision

9. Risk of impersonation (fraud)

10. Disincentive for appropriately trained personnel to accept deployment in rural settings

11. Cost of compensation for extra load

### Challenges encountered by respondents

The majority of respondents reported witnessing surgical task shifting in district hospitals (20/24 FGDs), and others said they’d seen it in private clinics (7/24 FGDs).

The following challenges were reported by a respondent who had participated in surgical task shifting:

“I have done several circumcisions. For the first time, I did it successfully but bleeding was the major problem. One of the bleeders was not ligated. So after discharging the patient, the area started bleeding. But the good thing he had my contacts and he called me. So I have learnt to do it with experience.”

Respondents reported fear resulting from the lack of sufficient knowledge, skills and experience, as well as the lack of supervision, legal cover, confidence, or equipment. Prescribing the wrong medicines was also mentioned.

The requirements for making surgical task shifting work are summarized below, as the availability of equipment and supplies, better remuneration, support supervision and training. Others included support from specialist surgeons and better operating room infrastructure.

### Requirements for surgical task shifting as named by clinical staff at Uganda hospitals

Availability of equipment and supplies

Salary increment / remuneration for the staff engaged in task shifting

Monitoring and evaluation

Support supervision specialized with ongoing training

Government & MOH support

Training by non specialist clinicians

Positive attitude of Surgeons to surgical task shifting

Funds to facilitate training

Willingness of health personnel to be trained

Infrastructure / Theatres

Source of data: key informant interviews in the Uganda task-shifting feasibility study 2012.

## Discussion

This study set out to explore perceptions of managers and frontline health workers on surgical task shifting across fifteen districts at 24 sites in Uganda. We found surgical task shifting was largely supported, although not without reservation. In any case, it was taking place at all the facilities we visited to some extent, even in the absence of guiding Ministry of Health policy.

Respondents largely understood the concept of surgical task shifting to include the passing on or delegation of a specified role from an appropriately-trained person in a higher cadre to a less-trained or less experienced cadre in the context of shortage of health workers. There were a few who did not grasp the meaning of the term and suggested it included referral from a higher level facility to a lower level facility in the context of lack of space, facilities or personnel at that particular time. There is no official policy framework that articulated the position of surgical task-shifting at the national level. At the global health level, WHO has issued guidelines encouraging appropriate delegation of tasks to lower cadre where it is safe and reasonable [11].

Even though surgical task shifting was largely supported, some respondents in our study said they often felt exposed or vulnerable when asked to take on tasks that are not in their legal scope of practice, especially in a situation where things could go wrong and result in a lawsuit or job dismissal. In the absence of regulation, some respondents said some clinical officers may abuse the practice by carrying out procedures away from their primary work stations where supervision is not possible. Further, the lack of policy may mean facility staff are asked to take on extraordinary tasks without the concomitant recognition or appropriate reward or job protections. These factors contribute to resistance to task shifting.

Resistance to task shifting occurs in settings where there is a lack of supervision and regulation. Lack of supervisor support leaves those engaging in task shifting with less on-site training for skill development. Some also expressed a sense of injustice; the officers delegating work get their time freed to go on to do other duties that may be more financially rewarding to them at the expense of the persons to whom less desirable tasks are delegated.

Other respondents worried the lack of proper documentation of these quasi-legal task-shifting operations leads to poor processes and outcomes. Efforts to formalize, and track task shifting must not only involve undertaking prospective studies but should also aim at improving the current health management information system to increase emphasis on surgical data collection.

Nearly all respondents acknowledged the need to meet the demand for surgical services that outpaces the capacity of health personnel whose scope of services clearly includes surgical procedures. Table 2 gives the sense of the scope and frequencies of procedures encountered in these study sites. There is recognition that other countries in the region, including Malawi, Mozambique and Tanzania, have had success with the practice [12-16].

There is recognition that other countries in the region, including Malawi, Mozambique and Tanzania, have had success with the practice [12-14]. Health workers in Uganda, however, pointed to a lack of country-specific evidence that surgical task shifting is feasible, sustainable and safe. There is also a lack of documentation of the surgical burden of disease in Uganda [6].

In all the facilities we visited, workers tended to support formalizing, supporting and scaling up surgical task shifting. Their recommendations are similar to those found by previous researchers; [9,17,18] that is, the long term success of task shifting hinges on serious political and financial commitments. These include a revised compensation scheme, reconfiguration of health teams, changed formal scopes of practice, regulatory frameworks and enhanced training infrastructures. The requirements articulated by the study participants as essential for moving task shifting forward to make it formal and safe.

What is clear is that surgical task shifting is currently practiced widely, even in the absence of regulation. Practitioners conceal the practice for fear of legal and professional consequences in the event of a poor outcome. Other barriers include lack of motivation to take on the extra load, poor work environments and a lack of space and equipment. These barriers have been articulated before, yet it is critical to pay attention to them [19].

The study demonstrates a willingness by managers and clinicians to formally embrace surgical task shifting with the caveats stated. This willingness is aligned with the realities Uganda faces, in the setting of a population of 33 million people with only 100 specialist surgeons who are mostly found in referral hospitals. Surgeons are rarely located in the rural communities where the majority of the population lives. Access to specialist care is further impeded by geographical distance, lack of appropriate means of transport and a mal-functioning referral system.

### Limitations of our study

The study sample represents only health facilities along the East-Central axis of the country. Due to logistical constraints, it was not possible to include informants from other parts of Uganda. Two sites visited did not have functional theatres at the time of the visit.

No reliable data on the safety of surgical task shifting was available from the facilities we visited. Uganda needs a well-designed prospective study in selected sites to establish the efficacy and safety of surgical task shifting. Considering that focus groups had different cadres participating in the same discussions, (juniors and their supervisors), some respondents could have withheld what would have otherwise been key or sensitive information for fear of negative consequences that could occur after the discussions.

In some instances focus group discussions we were interrupted by theatre staff being called to attend to emergencies cases.

Only one focus group discussion was conducted in each site.

### Recommendations

We recommend the Ministry of Health engage all stakeholders in developing formal surgical task shifting policy guidelines. The policy should address barriers such as resistance from health professionals, low salaries, and poor working conditions. Training and close supervision should be provided to all personnel who are asked to perform surgical procedures for which they did not receive pre-service training. The nation's health management information system should closely monitor who performs surgeries and what the outcomes are. All health personnel should receive health insurance coverage. As surgical task shifting is a response to a weak and understaffed health system, we recommend strengthening health system infrastructure, including workforce. This would include reducing workload, improving recruitment and retention through salary improvements, and improving working conditions. It would be helpful to Uganda and other low-income country settings to document successful examples of surgical task shifting.

## Conclusion

Surgical task shifting was strongly supported by facility managers and frontline health workers. Surgical task shifting is informally practiced widely, with varied understanding of the principles. Formal guidelines are absent. Uganda is ripe for a task shifting support program with formal training and supervision. There is also sufficient data on which to base a policy framework.

## Competing interests

The authors declare that they have no competing interests.

## Authors’ contributions

SL, GB, SB, MG originated concept. MG, SK, PS, OK, AK and SM collected data and participated in analysis. GM wrote the first draft. All authors performed reviews of drafts for intellectual content. All authors read and approved the final manuscript.

## Pre-publication history

The pre-publication history for this paper can be accessed here:

http://www.biomedcentral.com/1472-6963/13/292/prepub
